# Hand rheumatoid nodules

**DOI:** 10.1093/omcr/omae142

**Published:** 2024-11-25

**Authors:** Pierre Delzongle, Marie Binvignat, Christelle Nguyen

**Affiliations:** Service de Rééducation et de Réadaptation de l’Appareil Locomoteur et des Pathologies du Rachis, Hôpital Cochin, AP-HP.Centre-Université Paris Cité, 27 Rue du Faubourg Saint-Jacques, Paris 75014, France; Service de Rééducation et de Réadaptation de l’Appareil Locomoteur et des Pathologies du Rachis, Hôpital Cochin, AP-HP.Centre-Université Paris Cité, 27 Rue du Faubourg Saint-Jacques, Paris 75014, France; Service de Rééducation et de Réadaptation de l’Appareil Locomoteur et des Pathologies du Rachis, Hôpital Cochin, AP-HP.Centre-Université Paris Cité, 27 Rue du Faubourg Saint-Jacques, Paris 75014, France; Faculté de Santé, UFR de Médecine, Université Paris Cité, 15 Rue de l’École de Médecine, Paris 75006, France; INSERM UMR-S 1124, Toxicité Environnementale, Cibles Thérapeutiques, Signalisation Cellulaire et Biomarqueurs (T3S), Campus Saint-Germain-des-Prés, 45 Rue des Saints-Pères, Paris 75006, France

A 78-year-old woman had rheumatoid arthritis that was diagnosed when she was 17 years old. She received various medications including nonsteroidal anti-inflammatory drugs and gold salt therapy and was currently treated with methotrexate 10 mg weekly and low-dose oral corticosteroids. For the past two decades, she developed extensive subcutaneous rheumatoid nodules on both hands, totaling up to 40 nodules (Fig. 1). No other extra-articular manifestations, notably pulmonary involvement, were observed. Radiographs of both hands showed subluxations in the metacarpophalangeal and proximal interphalangeal joints, alongside carpal destruction (Fig. 2). Despite the disease activity was low, the patient reported high levels of hand-specific activity limitations, leading to her admission to a hand rehabilitation program.

**Figure 1:**
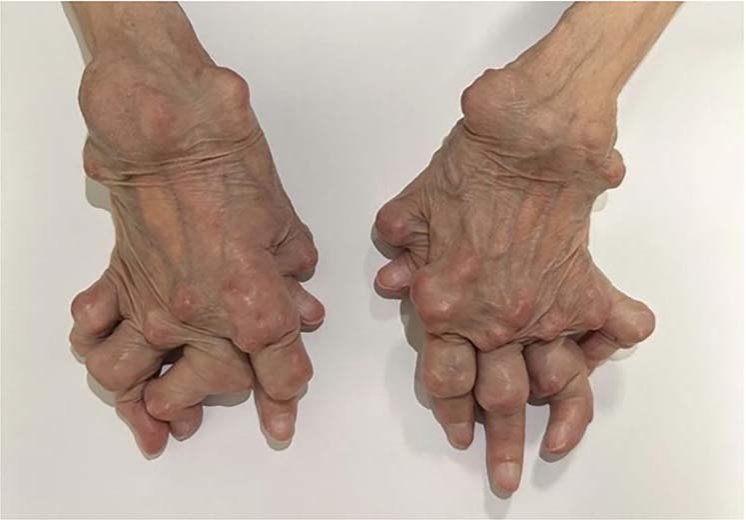
Photograph of both hands revealing multiple nodules on the dorsal aspect of the hands, involving the extensor tendons of the wrists and fingers.

**Figure 2:**
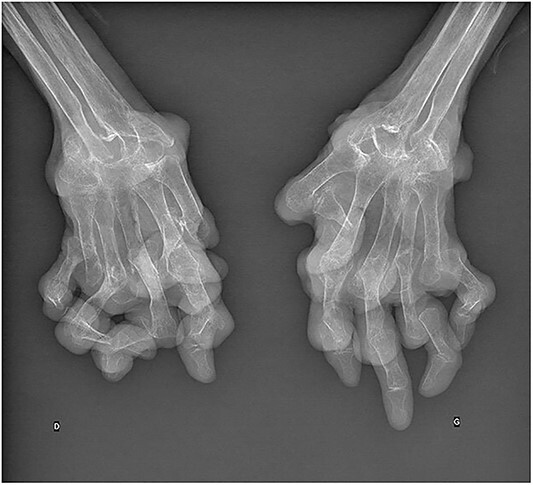
Radiographs of both hands showing subluxations in the metacarpophalangeal and proximal interphalangeal joints, alongside extensive carpal destruction.

Rheumatoid nodules are the most prevalent extra-articular manifestation occurring in up to 40% of individuals with rheumatoid arthritis and are most often observed in late stages of the disease.

## Conflicts of interest

None.

## Funding

None declared.

